# 2074. Longitudinal wastewater surveillance for endemic respiratory viruses and its correlation with clinically confirmed cases in Calgary, Canada

**DOI:** 10.1093/ofid/ofad500.144

**Published:** 2023-11-27

**Authors:** Kristine Du, Nicole Acosta, Barbara Waddell, Maria Bautista, Janine McCalder, Aito Ueno, Sudha Bhavanam, September Stefani, Carolyn Visser, Chloe Papparis, Puja pradhan, Lance Non, Paul Montesclaros, Imesha Perera, Jennifer Van Doorn, Kashtin Low, Kevin Xiang, Leslie Chan, Laura Vivas, Judy Qiu, Tiejun Gao, Rhonda Clark, Danielle Southern, Tyler Williamson, John Conly, Xiao-Li Pang, Bonita Lee, Steve Hrudey, Kevin Frankowski, Casey RJ Hubert, Michael Parkins

**Affiliations:** University of Calgary, Calgary, AB, Canada; University of Calgary, Calgary, AB, Canada; University of Calgary, Calgary, AB, Canada; University of Calgary, Calgary, AB, Canada; University of Calgary, Calgary, AB, Canada; University of Calgary, Calgary, AB, Canada; University of Alberta, Edmonton, Alberta, Canada; University of Calgary, Calgary, AB, Canada; University of Calgary, Calgary, AB, Canada; University of Calgary, Calgary, AB, Canada; University of Calgary, Calgary, AB, Canada; University of Calgary, Calgary, AB, Canada; University of Calgary, Calgary, AB, Canada; University of Calgary, Calgary, AB, Canada; University of Calgary, Calgary, AB, Canada; Mount Royal University, Calgary, Alberta, Canada; University of Calgary, Calgary, AB, Canada; University of Calgary, Calgary, AB, Canada; University of Calgary, Calgary, AB, Canada; University of Alberta, Edmonton, Alberta, Canada; University of Alberta, Edmonton, Alberta, Canada; University of Calgary, Calgary, AB, Canada; University of Calgary, Calgary, AB, Canada; University of Calgary, Calgary, AB, Canada; University of Calgary, Calgary, AB, Canada; University of Alberta, Edmonton, Alberta, Canada; University of Alberta, Edmonton, Alberta, Canada; University of Alberta, Edmonton, Alberta, Canada; University of Calgary, Calgary, AB, Canada; University of Calgary, Calgary, AB, Canada; University of Calgary, Calgary, AB, Canada

## Abstract

**Background:**

Wastewater (WW)-based surveillance of SARS-CoV-2 is an established tool for COVID-19 pandemic monitoring, providing a leading indicator to cases and hospitalizations. However, its potential for monitoring endemic respiratory viruses has not been elucidated. We assessed the occurrence of Influenza A (IAV), Influenza B (IBV), and Respiratory Syncytial Virus (RSV) RNA in WW treatment plants (WWTP) in Alberta's largest city and its correlation with clinical disease.

**Methods:**

Twenty-four-hour composite WW samples were collected weekly from three WWTP in Calgary between Mar'22/Apr'23. WW was concentrated and RNA extracted using the 4S-Silica Column. Viral RNA was quantified using a commercial TaqMan assay (IAV, Assay ID Vi99990011 po, Applied Biosystems) and established RT-qPCR assays targeting: the hemagglutinin gene (IBV) and nucleocapsid gene (RSV). Flow rates at each WWTP were used to create a composite city-wide metric for each target. WW values were compared to clinical data reported by Alberta Health Services and reported as total cases and test positivity rates across the Calgary Zone (or entire Province if granular data was unavailable).

**Results:**

IAV peaked in Calgary's WW between November-December 2022, IBV between February-April 2023 and RSV between November 2022-February 2023 (Figure 1,2,3). The composite-IAV signal positively correlated with weekly confirmed clinical cases within the Calgary Zone (Spearman's *r*= 0.83, p< 0.0001 for signals recorded the same week, and *r*= 0.85, p< 0.0001 for the prior week). The positive correlation existed regardless of influenza typed as H3N2, H1N1 or untyped. Furthermore, specimen test positivity rates across the entire province correlated with Calgary's WW measured IAV (Spearman's *r*= 0.88, p< 0.0001). The IBV WW signal correlated with clinical cases (Spearman's *r*= 0.63, p< 0.0001) and test positivity rates (Spearman's *r*= 0.74, p< 0.0001) across the entire province. Calgary's RSV WW correlated with clinical cases (Spearman's *r*= 0.56, p=0.001) and test positivity rates (Spearman's *r*= 0.50, p=0.007) across Alberta.

**Figure 1.**
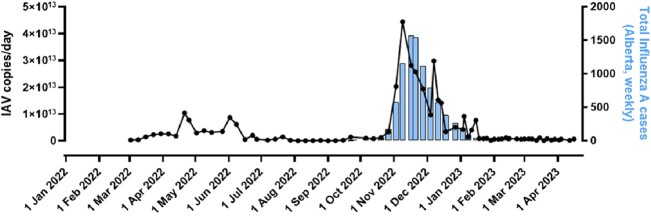
Comparison of aggregate WW IAV in Calgary compared to locally confirmed clinical cases.

**Figure 2.**
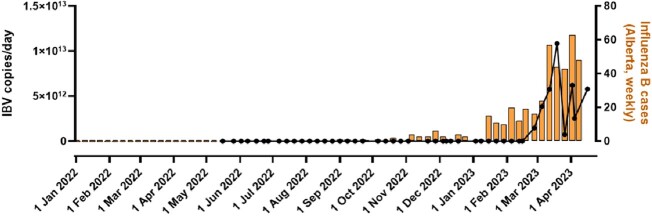
Trend of aggregate WW IBV signal in Calgary compared to clinically confirmed cases.

**Figure 3.**
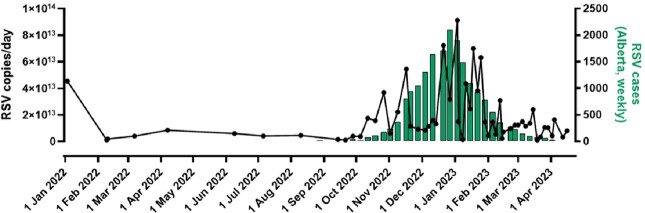
Trend of aggregate signal in Alberta for RSV compared to clinically confirmed cases over time measured.

There is a peak in the RSV signal from November 2022 to February 2023, with a correlation to Alberta’s weekly confirmed clinical cases.

**Conclusion:**

WW surveillance is an emerging technology enabling objective, unbiased and inclusive population-level monitoring of endemic respiratory viral infections.

**Disclosures:**

**All Authors**: No reported disclosures

